# Detection and Diagnosis of Retinoblastoma: Can Mobile Devices Be the Next Step Toward Early Intervention?

**DOI:** 10.7759/cureus.30074

**Published:** 2022-10-08

**Authors:** Abdur Rahman Jabir, Haniah A Zaheer, Myra A Zaheer, Eesha A Zaheer, Richard Birdsong

**Affiliations:** 1 School of Medicine, Northeast Ohio Medical University, Rootstown, USA; 2 School of Medicine, University of Pittsburgh Medical Center, Pittsburgh, USA; 3 School of Medicine, George Washington University School of Medicine and Health Sciences, Washington, D.C., USA; 4 Department of Pediatrics, Cleveland Clinic Foundation, Cleveland, USA; 5 Ophthalmology, Children's National Hospital, Washington, D.C., USA

**Keywords:** retinoblastoma, ocular oncology, pediatrics, ophthalmology, mobile application, mobile devices

## Abstract

Retinoblastoma (RB) is a common intraocular cancer in pediatric patients worldwide, and screening is routinely performed throughout the first few years of life. The diagnosis is often made clinically; however, the diagnosis can be delayed due to undetectable leukocoria because of small tumor size at the time of examination, missed appointments, non-compliance with eye examinations, or failure to perform the exam.

As mobile devices continue to gain in both popularity and functionality, their use via applications and smartphone attachments for ocular examination introduces a new avenue for screening, detection, and staging of RB both inside and outside the clinical setting. Currently, research regarding mobile device use is still in its infancy, and further research is required to determine whether mobile devices could play a significant role in assisting with the diagnosis of RB.

The purpose of this systematic review was to determine whether the existing literature supports the use of mobile devices by healthcare providers, specifically ophthalmologists and non-ophthalmologists, as well as by parents for the early detection of RB. A comprehensive literature search was conducted via PubMed, the Cumulative Index to Nursing and Allied Health Literature (CINAHL), and Web of Science with a total of 10 studies included in the final analysis.

## Introduction and background

Retinoblastoma (RB) is the most common intraocular cancer in pediatric patients worldwide, with an incidence of approximately one in every 16,000-18,000 live births, of which 200-300 cases are reported in the United States annually [1,2]. The five-year survival rate of RB reaches approximately 96% in the United States when detected early and treated promptly; however, in low- and middle-income settings globally, survival rates are significantly decreased due to lower overall detection and intervention resulting in the spread of RB [3,4]. Additionally, complications can occur with late detection of the disease, leading to a risk of invasion of surrounding structures (i.e., optic nerve, choroid, and anterior segment) and metastases to distal organs via the bloodstream [5,6]. Thus, early screening and diagnosis are pivotal in preventing visual loss and decreasing the risk of morbidity and mortality.

Diagnosis of RB is based on clinical presentation, physical examination, and diagnostic imaging. The most common clinical presenting sign is leukocoria. Other common findings include squint, proptosis, strabismus, nystagmus, orbital inflammation, and decreased vision [7,8]. The dilated fundoscopic exam is the gold-standard diagnostic method for visualizing nodular, white-colored retinal masses seen in RB [9]. While general physicians specifically look for normal red reflex on routine examination, delayed diagnosis can result from undetectable leukocoria due to small tumor size at the time of examination, missed appointments, non-compliance with eye examinations, or failure to perform the exam. Delayed diagnosis can lead to the progression of RB and worsening developmental outcomes in pediatric patients. Mobile devices provide a potential solution for both providers and parents to screen patients through applications and attachments to help eliminate the limitations that lead to missed diagnoses.

Current literature has examined the use of smartphone photography, smartphone applications, and attached devices in the diagnosis and detection of various ophthalmologic disorders such as diabetic retinopathy, glaucoma, cataracts, and amblyopia. However, no review on the use of mobile devices alone as a screening method for RB has been published to date. The goal of this study is to provide a comprehensive and up-to-date review of the available literature investigating the use of mobile devices by ophthalmologists, non-ophthalmologists, and/or parents to determine whether this utilization of mobile devices and associated applications or devices plays a role in screening RB.

## Review

Methods

**Figure 1:**
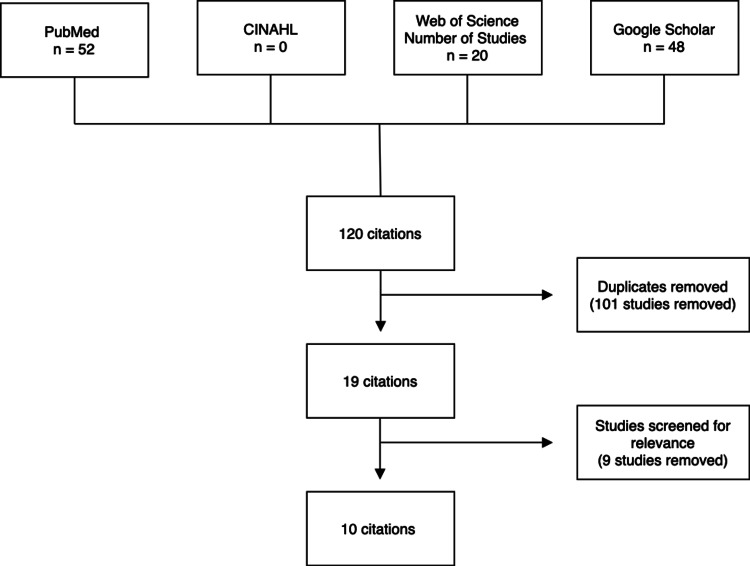
Flow diagram depicting literature search CINAHL: Cumulative Index to Nursing and Allied Health Literature

A systematic review was conducted by two authors (AR.J. and H.A.Z.) in August 2022. Keywords related to the diagnosis and screening of RB using mobile devices were used to search the following databases: PubMed, Web of Science, the Cumulative Index to Nursing and Allied Health Literature (CINAHL), and Google Scholar. Keywords used were as follows: mobile phone retinoblastoma; mobile applications retinoblastoma; smartphone retinoblastoma; mobile attachment retinoblastoma; smartphone detection retinoblastoma, mobile phone detection retinoblastoma; smartphone diagnosis retinoblastoma; mobile phone diagnosis retinoblastoma. Articles across the three databases were compiled and duplicate articles were removed based on titles. The remaining articles were first screened by title and abstract for relevance. If the relevance of the articles was not clear, then the full text was analyzed. The studies that were ultimately included in this review were published from 2013 to 2020 (Figure 1, Table 1).

**Table 1:** Inclusion and exclusion criteria for study selection RB: retinoblastoma

	Criteria
Inclusion criteria	The study must be in English. The study must involve only human subjects. The study must involve the use of mobile devices to detect RB.
Exclusion criteria	The study did not clearly state that mobile devices were used. Mobile devices imaged the fundus to screen for a different ocular pathology (e.g., diabetic retinopathy, glaucoma)

Results

Out of a total of 120 articles reviewed, 101 were removed due to duplication across the three databases. Based on inclusion and exclusion criteria, 10 articles were included in the final review (Table 2). Of these 10 studies, six were conducted outside of the United States and four were carried out within the United States.

**Table 2:** Summary of articles

Authors	Article title	Category	Study findings
Vagge et al. [10]	Evaluation of a Free Public Smartphone Application to Detect Leukocoria in High-Risk Children Aged 1 to 6 Years	Mobile application	The ophthalmoscope was superior to CRADLE in the early detection of leukocoria and it was concluded that CRADLE cannot be considered a standalone alternative to the ophthalmoscope for children aged 1 to 6 years at this time
Munson et al. [11]	Autonomous Early Detection of Eye Disease in Childhood Photographs	Mobile application	CRADLE found evidence of leukocoria in 16 of the 18 children from the test group, detected far in advance of a clinical diagnosis. The authors concluded that CRADLE showed promise in the early detection of eye pathology, such as RB
Khedekar et al. [12]	Smartphone-Based Application Improves the Detection of Retinoblastoma	Mobile application	MDEyeCare application was not able to detect leukocoria very well in the early stages but had improved detection of later-stage RB without the use of pharmacological dilation or general anesthesia. CRADLE did not show improved detection of RB in early or late tumor stages
Vargas-Cuentas et al. [13]	System for the Early Detection of Retinoblastoma	Mobile application	A Retinoblastoma Detection System composed of a mobile application, system algorithm, and the web server was able to correctly identify 95% of healthy controls with no ocular pathology and 93.33% of individuals with evidence of leukocoria
Ademola-Popoola and Olatunji [14]	Retinal Imaging With Smartphone	Mobile photography and videography use	The authors found that in situations where an ophthalmoscope is inaccessible or trained personnel are not readily available, the use of smartphone photography may be helpful in providing clear retinal imaging as a substitute
Haddock et al. [15]	Simple, Inexpensive Technique for High-Quality Smartphone Fundus Photography in Human and Animal Eyes	Mobile videography use	This article concluded that the smartphone, Filmic Pro application, and 20D condensing lens together provided a cheaper and portable alternative to imaging the fundus in the clinic, operating room under general anesthesia, and emergency department
Pujari et al. [16]	Unmodified iPhone XS Max for Fundus Montage Imaging in Cases of Retinoblastoma	Mobile videography use	This study found that newer generation iPhones, such as the XS Max, can effectively capture fundus images more easily and without the need for additional phone accessories/attachments
Abdolvahabi et al. [17]	Colorimetric and Longitudinal Analysis of Leukocoria in Recreational Photographs of Children with Retinoblastoma	Mobile photography use	Using amateur photography from parents from various digital cameras, the authors concluded that RB tumors may show unique colorimetric patterns that could potentially aid in earlier detection
Patel et al. [18]	Smartphone-Based, Rapid, Wide-Field Fundus Photography for Diagnosis of Pediatric Retinal Diseases	Mobile attachments	RetinaScope was in agreement with the clinician’s diagnosis 96% of the time and thus may allow the benefit of images of the fundus with a 90-degree field of view without sacrificing image quality or diagnostic accuracy
Bhaduri et al. [19]	Smartphone Wide-Field Fundus Photography in Retinoblastoma With a Nasal Endoscope	Mobile attachments	The study demonstrated the feasibility of capturing retinal tumor images via nasal endoscopy attachments to smartphones and proposed a useful supplement to retinal drawings to assess and follow patients over time

Mobile Application Use

Four studies investigated the use of mobile applications in the diagnosis of RB. Vagge et al. [10] studied children aged one to six years who underwent a complete pediatric ophthalmological examination; 122 children (244 eyes) met their enrollment criteria and were evaluated in the current study. Children were screened using the CRADLE smart app on an iPhone 7. The purpose of the study was to determine whether a white-detector smartphone app (CRADLE) can be utilized as an effective screening methodology in the detection of leukocoria. This application contained an algorithm that was designed to automatically detect a white pupillary reflex in photographs or videos. Cycloplegic retinoscopy and fundus examinations were performed for about 30 minutes after participants received one or two drops of pediatric combination, consisting of 1% tropicamide and 2.5% phenylephrine. The two pediatric ophthalmologists involved in the study were masked to the photoscreener results. Nine of the 244 eyes analyzed had leukocoria caused by an amblyogenic cataract evaluated by a penlight or ophthalmoscope, one had retinopathy of prematurity stage 5, and one had RB. None of the nine eyes with amblyogenic cataracts were detected by CRADLE. Two eyes with leukocoria caused by bilateral RB were detected by the smartphone app. Thus, there were 11 false-negative and 0 false-positive results. The results indicate a limitation in CRADLE due to a 100% specificity but only a 15.38% sensitivity. The study concluded that CRADLE cannot be considered an alternative to the ophthalmoscope for children aged one to six years.

Munson et al. [11] also investigated the ability of CRADLE to detect leukocoria in pediatric populations. This study retrospectively examined childhood digital photographs using CRADLE to determine whether there was evidence of leukocoria. Photographs of children who had been diagnosed with an eye pathology (bilateral RB, unilateral RB, bilateral cataracts, Coats disease, amblyopia, bilateral hyperopia) served as the test group while those without an eye pathology acted as the control group. The authors sought to evaluate the sensitivity, specificity, and accuracy of this application in order to determine whether it would be a valuable supplement to current protocols for leukocoria screening already in place. This application was downloaded onto the mobile devices of subjects and then searched through personal photographs stored on the phone for evidence of leukocoria. The study retrospectively assessed the photographs to determine if CRADLE would have detected leukocoria before the clinical diagnosis was made. The results showed that CRADLE found evidence of leukocoria in 16 of the 18 children from the test group and on average this would have been detected 484 days before the clinical diagnosis was made. Overall, the authors concluded that mobile applications such as CRADLE show promise in the early detection of eye pathology such as RB. However, the authors also noted the application’s detection of physiologic leukocoria as pathologic lowered the specificity and accuracy and presented as a limitation; therefore, further research using CRADLE is warranted.

A study by Khedekar et al. [12] looked into the ability of two smartphone applications to detect RB. The researchers investigated two applications - MDEyeCare and CRADLE - that were designed to detect leukocoria. The patients were initially screened by the MDEyeCare application and then later by the CRADLE application after clinical examination. Subjects of this study included individuals with either unilateral RB, bilateral RB, or no diagnosis of RB. The tumors were assessed based on size, location, and vitreous seeds; the individuals were then classified into groups A, B, C, D, or E based on their tumor grade. The rate of correct detection with the MDEyeCare application was 0% for group A, 50% for group B, 83% for group C, 100% for group D, and 100% for group E. These results showed that the application was not able to detect leukocoria very well in the early stages (groups A, B, and C) but improved in the detection of late-stage (group D and E) RB. Using the CRADLE application, the authors found that only four of the late-stage tumors were detected. Based on these results, the authors concluded that MDEyeCare was able to improve the rate of detection of late-stage RB without the use of pharmacological dilation or general anesthesia.

Vargas-Cuentas et al. [13] proposed an RB detection system that comprised a mobile application, an algorithm that detected leukocoria, and a web server. The study consisted of 15 subjects between the ages of six months and 18 years with RB and 20 healthy subjects between the ages of 18 and 45 years with no ocular pathology. The Retino application collected patient information and a picture of the patient’s face. The algorithm then used this information and adjusted the image to detect a bright pupil, which the authors stated was evidence of leukocoria. The final component of this system was the web server where patient data were stored. The results found that this system was able to correctly identify 14 of the 15 photos that showed evidence of leukocoria and 19 of the 20 healthy controls. Overall, the authors concluded that this system provided a simple and cost-effective approach for early identification of RB and may serve as a valuable alternative in areas where there is limited access to hospitals and medical professionals.

Mobile Photography and Videography Use

Four studies explored the use of mobile phones as a means for photography and videography of the pupil. Ademola-Popoola and Olatunji [14] conducted a study to determine the effectiveness of using a smartphone for retinal imaging. The authors proposed that smartphone photography may be useful in resource-limited economies or areas where access to an ophthalmoscope may not be readily available or affordable. In this study, the photography and videography function of the smartphone was used along with a noncontact 20D lens to capture clear images of the fundus after dilating the eye. The results showed that photos taken by the smartphone provided a clear visual of the fundus and the relevant pathology in subjects suffering from RB and other eye conditions. Based on their findings, the authors concluded that in situations where an ophthalmoscope is difficult to obtain or trained personnel are not available, the use of smartphone photography may serve as a viable substitute for providing clear retinal imaging.

Haddock et al. [15] investigated the use of a smartphone (iPhone 4 or iPhone 5), smartphone application, and 20D condensing lens in the acquisition of fundus imaging. Additionally, the light source from the smartphone was also utilized to serve as an indirect ophthalmoscope. The smartphone application Filmic Pro (Cinegenix LLC, Seattle, WA; http://filmicpro.com/) allowed for control of focus, exposure, and light intensity during imaging. This application recorded a video of the fundus; the still images were then extracted and analyzed. A 20D condensing lens was also used to focus the light on the patient’s retina and a Koeppe lens was added if the patient was under anesthesia and could not keep their eyelid open. The authors found that this setup provided high-quality imaging of the fundus in multiple settings. Familial exudative vitreoretinopathy and partially treated RB were two ocular pathologies that this technique was able to provide clear fundus imaging of in children under general anesthesia. Two ocular pathologies (vasculitis and choroidal nevus) in adults presenting to the emergency department were also clearly visualized. The authors concluded the smartphone, application, and lens together provided a cheaper and portable alternative to imaging the fundus in the clinic, operating room under general anesthesia, and emergency department.

Pujari et al. [16] performed fundus montage imaging with the help of an iPhone XS Max to investigate pediatric cases of RB. The purpose of the study was to demonstrate the efficacy of capturing fundus images without smartphone attachments. In a two-year-old RB patient, the researchers conducted a routine examination. The smartphone camera (iPhone XS Max) recorded a one-minute video with a continuous flashlight, and 14 screenshots were obtained. Results indicated that newer generation iPhones, such as the XS Max, can effectively capture fundus images with greater accessibility and without the need for additional attachments. The study demonstrates that smartphones hold promising results and may have benefits in routine follow-up and ophthalmic examination.

Abdolvahabi et al. [17] explored the use of mobile phones to detect leukocoria in recreational photographs of children. The authors proposed that amateur photography taken by parents may provide evidence of recurrent leukocoria, a common early sign of RB. Nine children with RB and 19 controls were included in this study and over 7,000 photographs were analyzed. Various digital cameras were used to capture the photography, including the camera of a smartphone (Apple iPhone 4®). Using these photographs, the hue, saturation, and value of the pupils of the RB participants were compared to those of the control participants without RB to determine whether there were any colorimeter differences. The authors found that analyzing the colorimetric properties may reveal differences in pupils with leukocoria and thus facilitate earlier detection of RB. In one patient, the photographs showed evidence of leukocoria as early as 12 days old, which was several months before the parents had noticed the leukocoria. In addition to earlier detection, these properties may also provide insight into disease progression through longitudinal follow-up of patients. Overall, the authors concluded that RB tumors may show unique colorimetric patterns that could aid in earlier detection; however, further research is warranted.

Mobile Attachments

Patel et al. [18] investigated whether a handheld smartphone-based fundus photography device, RetinaScope, had diagnostic value in detecting retinal disease in children. This device was able to capture the peripheral retina in five different fields of view: central, nasal, temporal, inferior, and superior. A video-capturing method was also implemented within the device in order to record the fundus in young children who were not able to cooperate with being photographed and spontaneously shifted their gaze. Retina specialists used these images and videos to first determine whether pathology was present and then reached a diagnosis on what they believed the pathology to be. This study looked at whether the diagnosis of ocular pathology made via the RetinaScope devices agreed with the clinical diagnosis. The results of this study found that both specialists were able to correctly identify all the pathological eyes as abnormal using this device. Additionally, they found that there was 96% agreement between the clinician’s diagnosis and the diagnosis made by the smartphone-based photography device. The authors concluded that RetinaScope may provide a unique benefit by capturing images of the fundus with a 90-degree field of view without sacrificing image quality or diagnostic accuracy.

Bhaduri et al. [19] investigated fundus images in RB patients with a nasal endoscope technique. Due to the expensive nature of traditional nasal endoscopy, a cost-effective approach was explored by attaching a nasal endoscope to a smartphone camera. The image was obtained via a smartphone and a conventional light source connected to the nasal endoscope to provide transpupillary illumination. Images of the fundus were acquired by placing the endoscope in front of the cornea. The combination of these devices was used to visualize the fundus of a patient with RB under anesthesia. Images of tumors, subretinal seeds, and the peripheral retina were obtained and observed. The overall field of view per image was approximately 60 degrees. The study demonstrated the feasibility of capturing retinal tumor images via nasal endoscopy attachments to smartphones and proposed a valuable supplement to retinal drawings to help in detecting tumors and following patients with RB over time.

Discussion

The current literature has shown promising results regarding the use of smartphones in the screening of RB. The use of mobile devices in the screening of RB can be broadly categorized into three domains: mobile applications, photography and videography, and mobile attachments (Table 3).

**Table 3:** Mobile devices, attachments, and applications used in the detection of retinoblastoma

Device or application	User	Mechanism of use	Studies	Advantages	Limitations
CRADLE	Any individual with a smartphone	Mobile application	Vagge et al. [10]; Munson et al. [11]	High specificity (100%); easy to use with clear instructions; detects leukocoria using pre-existing photographs	Low sensitivity; high rate of false negative results; low rate of detection of early-stage tumors
MDEyeCare	Any individual with a smartphone	Mobile application	Khedekar et al. [12]	Portable; easy to use with clear instructions	False negatives (i.e., incorrect red reflex or no reflex observed) and false positives (i.e. pseudoleukocoria) present
Retinoblastoma detection system	Any individual with a smartphone	A three-part system with a mobile application, web server, and system algorithm	Vargas-Cuentas et al. [13]	A cost-effective alternative in resource-limited regions; simple to use with clear instructions	Images are subject to misinterpretation
Fundus photography and/or videography	Ophthalmologist or trained medical professional	Standalone smartphone camera or smartphone camera paired with accessory lens	Ademola-Popoola and Olatunji [14]; Haddock et al. [15]; Pujari et al. [16]; Abdolvahabi et al. [17]	A simple method that provides high-quality images using high-resolution smartphone cameras; inexpensive; portable	Need for pupillary dilation for good image quality; fundus exam may need to be performed under anesthesia in young children; not easy to use for caregivers and non-medical professionals; images subject to potential misinterpretation
RetinaScope	Any individual with a smartphone	A smartphone-based fundus photography device	Patel et al. [18]	Videography easier to use in pediatric patients; portable; 90-degree field of view; high sensitivity	Need for pupillary dilation for good image quality; fundus exam may need to be performed under anesthesia in young children; not easy to use for caregivers and non-medical professionals; Images subject to potential misinterpretation
Nasal endoscope	Ophthalmologist or trained medical professional	Nasal endoscope attachment to a smartphone	Bhaduri et al. [19]	Cheaper alternative to traditional nasal endoscopy; portable	The technique requires two people; evidence of artifactual ring reflex due to reflection from the light source

The increased use of smartphones worldwide makes applications such as CRADLE and MDEyeCare particularly useful. Photography has been incorporated into screening patients inside and outside of a clinical setting for RB. Photography and videography can easily detect leukocoria in patients and have shown promise in obtaining fundus images in pediatric patients. Due to the difficult nature of performing pediatric eye exams, the use of videography can be particularly useful in obtaining images of the retina in pediatric patients who are difficult to examine and would otherwise need an exam under anesthesia. Videography is especially useful, as providers can record and select specific images from the video that are most optimal for viewing the fundus.

While indirect ophthalmoscopic examinations and imaging modalities, such as fundoscopy, are commonly used to diagnose RB, various factors such as cost, expertise, and need for pupillary dilation or general anesthesia may pose obstacles to obtaining consistent screening. The use of mobile phones and associated attachments, like the nasal endoscope, provide a cost-effective and convenient alternative that may overcome many of these barriers.

Study limitations

As the functionality of technology broadens, mobile devices and their associated applications may inversely become less user-friendly. Studies have shown a decreased proficiency in the use of mobile devices among individuals of older age, lower income, lower education, rural residents, and older adults in minority communities [20]. As a result, it is critical that patients using devices are provided with clear instructions and education on proper use, technique, and interpretation if not otherwise incorporated within the device or application.

Although much of the technology currently available to screen for RB is primarily used by healthcare providers, expanding these resources to family members may prove valuable in terms of facilitating early detection. Two studies included in this review explored the use of a 20D accessory lens in conjunction with a smartphone camera to capture a fundus image and found that this may provide a cheaper and more portable alternative to traditional fundoscopy [14,15]. The feasibility of accessory lens use by family members can also be seen as a limitation. A recent publication of fundal images taken by family members found that after less than 10 minutes of training, family members were able to capture adequate imaging of the fundus using an accessory lens [21]. Thus, there may be promise in the use of an accessory lens, as it can be taught relatively quickly; however, continued education on proper technique and increased accessibility of these accessories for routine use by parents for RB screening remain an area with room for improvement.

An additional user limitation that must be considered involves application availability in multiple languages. Patients with low English language proficiency may also be at a disadvantage if the translation is not made available. Both CRADLE and MDEyeCare are available for English and Spanish speakers; however, it will be beneficial to make these applications available in other languages as well. Overall, CRADLE and MDEyeCare provide a useful screening method for leukocoria. While Khadekar et al. [12] found MDEyeCare particularly helpful in patients who face barriers to seeing a healthcare provider, MDEyeCare relies on subjective interpretation. CRADLE, on the other hand, displays the result of the image as a “normal” or “white” eye, eliminating the need for interpretation. This is an important factor to consider, as parents and families should rely more on using applications that incorporate interpretation into their algorithms.

Although it is important to provide proper education and warning symptoms for RB, it is equally important to reassure parents about false-positive signs and false-negative signs. A false-positive result occurs in the setting of photoleukocoria or any other situation where the pupil appears white despite no ocular pathology. The phenomenon of photoleukocoria occurs when the flash from a camera causes the pupil to appear white despite the individual having no ocular pathology. Asensio-Sánchez et al. [22] have described a case study where a three-year-old male presented with suspected leukocoria in the right eye. However, a normal ocular examination was able to rule out any true indication of RB, and it was determined that off-axis photography of the pupil had captured an image of the optic nerve and resulted in a false diagnosis of leukocoria in this case. To avoid this, caregivers should be instructed on how to orient the child when taking photographs to accurately capture the pupil. A false-negative result presents as a red reflex or no reflex despite a tumor being present. Thankfully, applications such MDEyeCare are designed to automatically correct variables that commonly affect the reliability of standard smartphone photography; however, this limitation remains. While the incidence of false positives and false negatives is lower compared to standard photography, before being implemented as a diagnostic tool alone, application-based interpretation should have further clinical confirmation.

Overall, the heterogeneity of the studies included in this review and the varying types of mobile devices and accessories used to image the retina also present a limitation. In 2013, Abdolvahabi et al. described the use of an iPhone 4 to capture digital images of the retina, while Pujari et al. later investigated the use of an iPhone XS Max to capture images of the fundus. This dissimilarity between the types of devices used makes it difficult to compare and draw conclusions across these studies, as the imaging capabilities and quality of these mobile devices differ.

Lastly, many of these studies have low statistical power due to their small sample sizes; therefore, it may be difficult to determine the true effect of mobile devices and definitive conclusions cannot be made until further research is conducted.

Future directions

The use of mobile devices in the diagnosis of other ocular pathologies may help to guide future studies investigating RB. For example, in the diagnosis of diabetic retinopathy, a meta-analysis by Tan et al. [23] found that smartphone ophthalmoscopy performed well and may provide a viable alternative in areas where expensive retinal equipment is not available. Another study by Sengupta et al. [24] compared the sensitivity and specificity of smartphone-based fundus imaging to conventional fundus photography and clinical exam. The authors found high sensitivity and specificity among the diagnostic methods, good agreement between graders, and fewer low-quality images when using the smartphone setup. In the diagnosis of glaucoma, Guo et al. [25] found that a mobile application lowered an individual’s glaucoma burden by allowing for real-time diagnosis and access to reliable screening from the convenience of their smartphone. As research continues to find mobile devices helpful in the diagnosis of other ocular pathologies, further investigation into their use in the context of RB shows promise as a method of screening that can be translated to clinical practice.

An additional method that can be further investigated could involve the use of orbital ultrasound as a mobile device attachment. Orbital ultrasound has been a valuable tool in obtaining a more detailed visualization of structures in the eye [26]. A-scans can measure spike height, regularity, reflectivity, and sound attenuation, making it easier to characterize internal tumor structures and composition [26]. B-scans allow for visualization of the anatomic location, shape, border, and size of lesions and can be used for anterior segment, peripheral retina/vitreoretinal, and choroidal pathology [26]. The use of ultrasound in the diagnosis of RB has also been commonly employed, and multiple studies have shown how this imaging modality can aid in detecting tumor calcification [27,28]. A study by Finger et al. [27] found ultrasound to be valuable in the diagnosis of RB by taking advantage of the unique ability of ultrasonography to capture the calcified tumor from various angles and views that are not possible with other imaging modalities. Kendall et al. [28] showed that the attenuation of sound waves and bright echoes posterior to the tumor was easily visible on ultrasound and allowed for clear detection. The use of ultrasounds as a mobile device attachment may be useful to explore in settings where equipment, such as an ophthalmoscope, is unavailable and quick visualization is necessary. While the value of ultrasound has already been shown, the addition of a smartphone attachment may provide an easily accessible technique that is more portable and provides improved resolution. Pairing a portable ultrasound device with a smartphone has been investigated in other fields of medicine and many of these findings may be helpful in the diagnosis and management of RB [29,30].

Attending follow-up appointments has been difficult for many patients with ocular pathologies during the coronavirus disease 2019 (COVID-19) pandemic [31-33]. During the pandemic, many families have found it more difficult to make or attend appointments, increasing the risk of delayed eye exams and the progression of disease in patients with RB. Social determinants of health also pose a limitation to gaining regular access to healthcare. Thus, mobile devices may be of more use in patients who are unable to leave their homes due to socioeconomic barriers or isolation due to the pandemic, making proper education on what RB is, how to detect leukocoria, and tools for early detection that much more important.

## Conclusions

The literature to date suggests that the use of mobile devices in the context of applications, photography, videography, and attached ophthalmologic devices have the potential to serve as a screening tool for RB; however, further research is required before this can be implemented as a standardized practice by healthcare providers. There is further work to be done through education on the importance of detecting RB before mobile devices can have a substantial effect on patient outcomes. Additional studies, particularly randomized controlled trials, would serve as an effective means to understand how mobile devices can further aid patients across various socioeconomic backgrounds and clinical settings.
